# Effect of dietary supplementation of lauric acid on growth performance, antioxidative capacity, intestinal development and gut microbiota on black sea bream (*Acanthopagrus schlegelii*)

**DOI:** 10.1371/journal.pone.0262427

**Published:** 2022-01-13

**Authors:** Sami Ullah, Jinzhi Zhang, Bingying Xu, Arnaud Fabrice Tegomo, Gladstone Sagada, Lu Zheng, Lei Wang, Qingjun Shao

**Affiliations:** 1 College of Animal Sciences, Zhejiang University, Hangzhou, China; 2 Ocean College, Zhejiang University, Zhoushan, China; 3 Ocean Academy, Zhejiang University, Zhoushan, China; Kafrelsheikh University, EGYPT

## Abstract

A feeding trial of eight weeks was conducted to examine the influence of food supplementation with lauric acid (LA) on *Acanthopagrus schlegelii* (juvenile black sea bream). A 24 percent fish meal baseline diet was created, while the other two diets were generated with dietary supplementation of graded points of LA at 0.1 percent and 0.8 percent, respectively. Each diet was given a triplicate tank with 20 fish weighing 6.22 ± 0.19 g. In comparison with the control group, the weight gain rate, growth rate, as well as feed efficiency of fish fed of 0.1 percent diet of LA were considerably (P < 0.05) greater. The total body and dorsal muscle proximate compositions did not change significantly between groups (P > 0.05). Triglyceride (TG) content was considerably (P < 0.05) greater in the LA-supplemented meals eating group in comparison with the control group. In the group eating LA-supplemented meals, the height of villus and the number of goblet cells/villus were considerably (P < 0.05) larger. The microbial makeup of the gut was also studied. The differences in phyla, class, and family level were not statistically significant (P > 0.05). Firmicutes in the phylum, Betaproteobacteri, Gammaproteobacteria, and Clostridia in the class, and Clostridiaceae in the family were all substantially increased with higher levels of LA supplementation (P < 0.05). According to the findings of this study, an LA-supplemented diet improves fish development, antioxidative capability, gut microbiota and intestinal health.

## Introduction

The aquaculture sector is one of the emerging sectors with an 8% average yearly growth rate. Fisheries and aquaculture almost provide 20% of protein worldwide and it is estimated that worldwide it provides 3.2 billion of fish demand [1]. In the current situation, fishmeal (FM) and fish oil (FO) around 68–88% comes from the aquaculture sector [2]. In aquaculture, the feeding habits of fish are affected by unsuitable farming conditions which make the environment unhealthy for the fishes [3]. Similarly, for intensive aquaculture systems, due to the limitation of natural feed sources, the growth of fish is affected which is covered by providing a balanced diet for the fish [4, 5].

Medium-chain fatty acids (MCFAs) are widely used in aquaculture as they are obtained from organic sources and provide the animals with readily available energy. MCFA has been used as an antibiotic supplement by Kuang, Wang [6]. Similarly, Medium-chain triglycerides (MCTs) can enhance growth performance in animals like weanling pigs [7]. MCFA is reported as a growth enhancer because it bears rapid digestive properties. The molecules can also pass through membranes without the expenditure of energy. They have obligatory oxidation which makes them acceptable for young animals as a feed source [8]. MCFA has antimicrobial effects as well as a protective effect on the intestinal microarchitecture previously observed in pigs [9]. MCFAs have been also used in metabolism [10], immune response and to increase tight junction permeability [10]. Dietary administration of acidifiers in fish feed controls the overgrowth of pH-sensitive pathogenic bacteria which further constrain the growth rate of various pathogenic bacteria and ultimately support the beneficial intestinal flora growth [11]. Other important members of MCFA families include caproic acid, caprylic acid and capric acid. LA (dodecanoic acid) with 12 carbon atoms is classed with the MCFAs group [12]. Soliva, Meile [13] identifies that LA as the most powerful of the MCFAs. Dohme, Machmüller [14] take LA for the most powerful MCFA in rumen Archaea which can suppress protozoal numbers.

LA is found in coconut oil (52% of the total 92% saturated fats) and plays an essential role in the healing of wounds [15]. Present in plant oils, fruits, seeds and breast milk and diverse action in various tissue like antimicrobial properties [16, 17]. LA is a potent antimicrobial agent among saturated fatty acids [18] which is reported in previous in vitro studies [18] suggesting that LA can be used as a substitute for other oils [19]. It was reported that LA can also exert its effect by inducing an immune response [20].

As earlier stated, coconut fatty acid-based sodium salt distillates, mainly rich in LA [C12], improved intake of feed, nutrient absorption and gut development as well as the fish’s growth rate [21]. In broilers, the addition of MCFA in the feed not only enhanced broiler health but also increased broiler growth [22]. LA can modulate the microbiome to enhance gut health, which can be further enhanced [23]. It remains stabilized while passing through the gastrointestinal tract that leads to its ultimate absorption [9, 18]. This property directly encounters LA with gut microbiota and helps in improving the host health and physiology with the help of improved metabolism and immunity [24]. LA is directly used in the formulation of animal feeds [8].

The Western Pacific Region is home to a large population of black sea bream. Its economic importance and aptitude for aquaculture are growing in favour in South East Asia. [25]. There has been minimal research into the nutrient needs of black sea bream [26, 27], however, complete knowledge about the composition of food of an *Acanthopagrus schlegelii* is still required. The current study’s main goal is to evaluate the dietary LA requirement for *Acanthopagrus schlegelii* fed a high soybean meal diet as a feed supplement. To the best of our knowledge, however, LA on *Acanthopagrus schlegelii* has not yet been documented. As a result, the current study looks at the impacts of LA growth performance, intestinal immune response, body composition, feed consumption, serum antioxidative capacity, and the gut microbiome.

## Materials and analysis methods

### Sample collection along with experimental conditions

The young black sea bream was obtained at the Zhejiang Province Marine Fisheries Research Institute in Zhoushan, China. To facilitate the feeding test before the experimental diet, the experimental fish were fed the control diet for 14 days. The experiment was conducted in triplicate. There were 400L fiberglass nine tanks. Each tank received twenty healthy fish (average weight per fish: 6.22 ± 0.19 g). Each tank was filled with sand-filtered and aerated seawater at a rate of 2 L/min. Water temperature (27 °C), pH (8.1–8.3), and salinity (26–29 g L^-1^) were all checked daily and kept constant throughout the experiment. The amount of dissolved oxygen level was kept above 5.00 mg L^-1^ during the feeding phase. For eight weeks, all of the fish were nourished twice a day (8:00 AM and 4:00 PM). The amount of feed consumed was tracked on an everyday basis. The feces of fish were collected on a morning basis from each tank at 6:00 AM starting the sixth week, before the following meal. The feces were collected using the procedures mentioned before [28] for further examination, the samples were frozen at -20°C.

### Experimental diets

The South China University of Technology provided lauric acid (LA). According to the *Acanthopagrus schlegelii* nutritional necessities, an isonitrogenous and isoenergetic (41.5% and 19.0 kJ g^-1^ respectively) diet was developed as a control (LA 0.0%). Various supplemented diets with a graded degree of LA were developed (0.1, and 0.8%). Fishmeal (FM), soybean meal (SBM), soy protein concentrate (SPC), and squid liver meal were the primary protein sources, while fish oil, maize oil, and soybean lecithin were the primary lipid sources (Table 1).

**Table 1:** Composition of basal diet and proximate investigation.

Constituents	Content (g kg^-1^)
FM^a^	199
SBM^b^	450
Soy protein concentrate	30
Squid liver meal	30
α-starch	70
Fish oil	30
Corn oil	58
Soy lecithin	20
Ca(H_2_PO_4_)_2_·H_2_O	20
CaCO_3_	7
Alpha cellulose	41.4
Vitamins^c^	7.5
Minerals^d^	7.5
Y_2_O_3_	1
Phytase	0.5
L-carnitine	2
CMC	5
Carrageenan	2
Methionine	8
Lysine	6.1
Taurine	5
Total	1000.0
Proximate composition^e^	
Crude protein	390.8
Lipid	145.1
Moisture	104.5
Ash	89.81

^a^Imported from Peru by Minghui Feed Company in Jiaxing, China. The crude protein content is 57.95%, while the crude fat content is 10.28%.

^b^Minghui Feed Company in Jiaxing, China provided the purchase. 42.87% crude protein; 4.64% crude lipid

^c^Vitamin premix (mg kg^-1^ of diet): retinyl acetate, 40; cholecalciferol, 0.1; DL-α-tocopheryl acetate, 80; menadione, 15; niacin, 165; riboflavin, 22; pyridoxine HCl, 20; thiamin mononitrate, 45; D-Ca pantothenate, 102; folic acid, 10; vitamin B_12_, 0.9; inositol, 450; ascorbic acid, 150; Na menadione bisulphate, 5; thiamin, 5; choline chloride, 320 and paminobenzoic acid, 50.

^d^Mineral premix (mg kg^-1^ of diet): Na_2_SiO_3_, 0.4; CaCO_3_, 350; NaH_2_PO_4_·H_2_O, 200; KH_2_PO_4_, 200; MgSO_4_·7H_2_O, 10; MnSO_4_·H_2_O, 2; CuCl_2_·2H_2_O, 1; ZnSO_4_·7H_2_O, 2; FeSO_4_·7H_2_O, 2; NaCl, 12; KI, 0.1; CoCl_2_·6H_2_O, 0.1; Na_2_MoO_4_·2H_2_O, 0.5; AlCl_3_·6H_2_O, 1; and KF, 1.

^e^Value for the proximate analysis of the test diets are means of triplicate analyses.

The dry ingredients were finely powdered using a 178 m mesh sieve. Gradually, fish oil, maize oil, along soybean were added in proper combination. The ingredients were homogenized by mixing and stirring properly, extruded via a pelletizer (1.2 mm module) after adequate mixing (Modle HKJ-218, HUARUI, Wuxi, China). The pellets that were extruded became steam-cooked for 10 minutes before being dried for 72 hours at 24 °C via an electric fan along with an air conditioner. The pellets were collected and kept in a 20 °C refrigerator.

### Sample collection along with analytical methods

Following the feeding trial at the end of the 56th-day, all experimentally observed fish were fasted for 24 hours and sedated with tricaine methanesulphonate (60mg L-1). Each fish’s body weight and length were measured. Initially, three fishes were selected randomly for whole-body composition investigation from each tank. Five fish were sampled with their whole intestines from each tank in a sterile setting and kept at -80 °C for microbiota analysis. The rest were utilized to collect blood, fat, dorsal muscles, liver and gut samples.

A needle of 27-gauge and 1 mL of the syringe were used to draw the blood sample from the caudal vein. The blood samples were then stable for 2 hours at 4 °C before being centrifuged (3000 rpm) for 15 minutes at 4 °C to extract serum. Furthermore, chemical analyses were carried out following the previously mentioned standard methodology [29]. For moisture determination, the grounded samples were dried at 105 °C in a forced air oven for 24 h. The crude protein was calculated using Kjeldahl-nitrogen factor 6.25. Using the Soxhlet extraction process, the crude lipid was extracted with ether for 6 hours. To determine the total ash content, samples were burned in a muffle furnace for 6 hours at 550 °C. Meanwhile, the total phosphorus concentration was assessed using the molybdovanadate technique [29], which involved weighing the ash, wet-digesting it with HNO3 and HCl, and then analysing it. Total protein (TP), total cholesterol (T-CHO), triglycerides (TG), aspartate aminotransferase (AST), alanine aminotransferase (ALT), albumin (ALB), catalase (CAT) activity, and total antioxidant capacity (T-AOC) activities (MDA) in serum, the activity of digestive enzymes (lipase, amylase, and trypsin) in the stomach, foregut, midgut, and hindgut were assessed using a diagnostic kit using the existing approach described by [29].

### Intestinal histomorphology (H&E)

For histomorphometric analysis, the intestinal samples were dried in increasing concentrations of ethyl alcohol, used xylene for washing, and finally, paraffin wax was used to cover the sample. The final sample was divided into six slices (each sliced of 6 μm in size), positioned on the slide of glass, and at last, stained with dyes like hematoxylin and eosin.

Three slides were made and evaluated by light microscopy for each intestinal segment morphological observation. This procedure was carried out at Zhejiang University’s animal physiology laboratory in Hangzhou, China. OLYMPUS (CX21) microscope was used to obtain the necessary pictures of prepared experimental samples. The villus height of twelve well-oriented villi per picture was measured using the Image-Pro Plus (IPP6.0) programme. From the villi tip to the villi crypt junction, the precise villus height was measured.

### Microbiota analysis

DNA extraction, PCR amplification, and Illumina MiSeq sequencing were all performed. Fecal samples were used for the extraction of total genomic DNA (n = 4) using a commercially available E.Z.N.A. ^®^Stool DNA Kit (D4015, Omega, Inc., USA). PCR (12.5L of Pusion Hot start flex 2X Master Mix, 2.5L of each primer, and 50 ng of template DNA) was used to amplify the V4-V5 sections of the bacterial 16S rRNA gene using universal primers 338F (5’ ACTCCTACGGGAGGCAGCAG 3’) and 806R (5’ GGACTACHVGGGTWTCTAAT 3’).

The PCR amplification products were detached on a 2% agarose gel electrophoresis, and the targeted fragments were re-gained using the AxyPrep PCR Cleanup Kit. The pooled library was put onto an Illumina MiSeq, and paired-end sequencing (2 300 bp) was performed using the MiSeq Reagent Kit V3 (600 cycles).

### Calculation and statistical analysis

The following equations were used to calculate growth performance and feed utilization:

Initial average body weight (IBW, g).
Final average body weight (FBW, g).
Weight gain rate (WGR, %) = 100 × (final body weight—initial bodyweight) / initial body weight.
Specific growth rate (SGR, %/day) = 100 × (ln final body weight—ln initial body weight) / days.
Mean feed intake (MFI, g fish^-1^ d^-1^) = air dry fed in g / (fish in g × day)
Feed efficiency (FE, %) = 100 × fish wet weight gain in g / air dry fed in g
Condition factor (CF, g cm^-3^) = 100 × [(final body weight in g) / (final body length in cm)^3^]
Hepatosomatic index (HSI, %) = 100 × (liver weight in g / body weight in g).
Intraperitoneal fat ratio (IPR %) = 100 × (intraperitoneal fat weight in g / body weight in g).
Viscerosomatic index (VSI, %) = 100 × (viscera weight / body weight);
Survival rate (SR, %) = 100 × (final fish number / initial fish number).

For all statistical studies, the SPSS software version 20 (SPSS Inc., Chicago, IL) was utilized. To evaluate the data for normality and homogeneity of variances, the Kolmogorov-Smirnov and Levine’s tests were used. To distinguish the data, one-way ANOVA was used, followed by Tukey’s test. The significance threshold was set at (P < 0.05), and the findings were reported as means standard deviations.

### Ethical statement

All of the fish were handled following the Administration of Laboratory Animals Guidelines released by the State Science and Technology Commission of China. The ZJU Committee for Experimental Animals Ethical Investigation granted the authorization number (ZJU20190052) for this.

## Results

### The rate of growth and the amount of feed consumed

Table 2 shows the feed utilization characteristics and growth results. LA supplemented diets had a substantial (P < 0.05) effect on specific growth rate (SGR), weight gain rate (WGR), final body weight (FBW), feed efficiency (FE), and hepatosomatic index (HSI). The LA 0.1 percent supplemented diets feeding group had the greatest SGR, WGR, FER, and HSI. In all treatment groups, there was no significant change in mean feed intake (MFI), condition factor (CF), or intraperitoneal fat ratio (IPR) (P > 0.05).

**Table 2:** The influence of dietary dose of lauric acid (LA) on juvenile black sea bream (*Acanthopagrus schlegelii*) growth rate, feed consumption, and morphometric parameters.

Parameters	Diets		
	Control (0.0)	LA 0.1	LA 0.8
IBW	6.23 ± 0.20	6.20 ± 0.13	6.22 ± 0.26
FBW	38.46 ± 0.44 ^**b**^	47.16± 3.03 ^**a**^	43.19 ± 1.37^**ab**^
WGR (%)	517.39 ± 14.70^**c**^	661.43 ± 3.96^**a**^	595.13 ± 26.88^**b**^
SGR (% per day)	3.25 ± 0.04^**c**^	3.62 ± 0.01^**a**^	3.44 ± 0.07^**b**^
MFI (g per fish day^-1^)	0.87 ± 0.04	0.99 ± 0.68	0.87 ± 0.06
FE (%)	75.38 ± 2.10^**b**^	82.13 ± 3.12^**a**^	81.61 ± 0.26^**a**^
CF (g cm^-3^)	2.75 ± 0.02	2.81 ± 0.07	2.80 ± 0.40
HSI (%)	1.70 ± 0.10^**b**^	2.27 ± 0.32^**a**^	0.87 ± 0.06^**c**^
IPR (%)	1.98 ± 0.25	2.72 ± 0.53	2.57 ± 0.36
VSI (%)	7.36 ± 0.36	8.55 ± 1.06	7.66 ± 0.53
SR (%)	100 ± 0.00	100 ± 0.00	98.33 ± 2.88

Note: Values are given as mean ± SD (n = 3) and each group show considerable difference (P < 0.05) within distinct superscripts.

Abbreviations: IBW, Initial average body weight; FBW, Final average body weight; WGR, weight gain rate; SGR, specific growth rate; MFI, mean feed intake; FE, feed efficiency; CF, condition factor; HSI, hepatosomatic index; IPR, intraperitoneal fat ratio; VSI, viscerosomatic index rate; SR, survival rate.

### Composition of the whole body and dorsal muscle

Table 3 shows the makeup of the total body and dorsal muscle. All dietary groups had non-significant differences in protein, fat, ash, and phosphorus composition of the total body (P > 0.05). While moisture in the control group was considerably in higher amount compared to the other groups (P < 0.05), Protein, lipid, and moisture levels in dorsal muscle were not substantially different across all dietary groups (P > 0.05), however, ash and phosphorus levels in the control group were considerably quite in higher amount compared to other present groups (P < 0.05).

**Table 3:** The influence of dietary levels of lauric acid (LA) on the proximate compositions of juvenile black sea bream (*Acanthopagrus schlegelii*) of total body and dorsal muscle.

Parameters	Diets		
	Control (0.0)	LA 0.1	LA 0.8
Whole body			
Protein	17.61 ± 0.34	17.75 ± 0.54	18.04 ± 0.29
Lipid	11.22 ± 0.44	13.56 ± 0.88	12.59 ± 1.44
Moisture	66.57 ± 0.40^**a**^	65.50 ± 0.55^**b**^	65.27 ± 0.27^b^
Ash	4.94 ± 0.16	5.08 ± 0.35	5.30 ± 0.37
Phosphorus	2.65 ± 0.13	1.85 ± 0.54	2.52 ± 0.50
Dorsal muscle			
Protein	20.92 ± 0.12	20.61 ± 0.36	20.44 ± 0.29
Lipid	4.05 ± 0.04	4.74 ± 0.74	4.37 ± 0.16
Moisture	73.69 ± 0.13	73.59 ± 0.89	73.58 ± 0.14
Ash	1.56 ± 0.02^a^	0.11 ± 0.04^**b**^	0.09 ± 0.01^**b**^
Phosphorus	1.06 ± 0.01^a^	0.05 ± 0.03^**b**^	0.05 ± 0.04^**b**^

Note: Values are given as mean ± SD (n = 3) and each group show considerable difference (P < 0.05) within distinct superscripts.

### Digestive enzymes activities

Digestive enzyme activities of stomach, foregut, midgut and hindgut are presented in Table 4. There was no significant difference (P > 0.05) in lipase, amylase, trypsin in the stomach, foregut, midgut and hindgut. While lipase in the foregut of the control group was significantly higher as compared to other present groups, (P < 0.05).

**Table 4:** Influence of dietary levels of lauric acid (LA) on digestive enzyme activities of the juvenile black sea bream (*Acanthopagrus schlegelii*).

Parameters	Diets		
	Control (0.0)	LA 0.1	LA 0.8
Stomach			
Lipase (U gprot^-1^)	1.13 ± 0.12	0.87 ± 0.15	1.08 ± 0.04
Amylase (U mgprot^-1^)	0.90 ± 0.22	0.97 ± 0.07	0.94 ± 0.23
Foregut			
Lipase (U gprot^-1^)	1.53 ± 0.17^**a**^	0.98 ± 0.22^**b**^	1.14 ± 0.19^**ab**^
Amylase (U mgprot^-1^)	1.63 ± 0.27	1.84 ± 0.19	2.10 ± 0.08
Trypsin (U mgprot^-1^)	129.42 ± 46.05	111.47 ± 12.81	115.86 ± 22.31
Midgut			
Lipase (U gprot^-1^)	1.75 ± 0.24	1.61 ± 0.02	2.01 ± 0.23
Amylase (U mgprot^-1^)	2.85 ± 0.77	2.36 ± 0.03	2.49 ± 0.17
Trypsin (U mgprot^-1^)	226.01 ± 24.47	109.12 ± 2.91	182.03 ± 67.86
Hindgut			
Lipase (U gprot^-1^)	2.41 ± 0.51	2.18 ± 0.11	2.18 ± 0.13
Amylase (U mgprot^-1^)	2.58 ± 0.08	2.76 ± 0.27	2.45±0.54
Trypsin (U mgprot^-1^)	231.28 ± 73.36	255.44 ± 3.82	201.99 ± 38.14

Note: Values are given as mean ± SD (n = 3) and each group show considerable difference (P < 0.05) within distinct superscripts.

### Serum biochemical, immune and antioxidant indicators

Table 5 shows serum biochemical, immune, and antioxidant markers. The serum TP, T-CHO, AST, ALT, and ALB levels were not substantially (P > 0.05) altered by any of the dietary categories. Overall, higher concentrations of LA supplementation raised TG (P < 0.05). The LA-supplemented meals did not affect serum immunological and antioxidant markers of CAT and T-AOC activity (P > 0.05) While MDA of the control group was significantly higher as compared to other present groups, (P < 0.05).

**Table 5:** Influence of dietary levels of lauric acid (LA) on serum biochemical index, immunological and antioxidant indices of juvenile black sea bream (*Acanthopagrus schlegelii*).

Parameters	Diets		
	Control (0.0)	LA 0.1	LA 0.8
TP (g L^-1^)	41.30 ± 2.89	46.48 ± 5.81	48.45 ± 4.29
T-CHO (mmol L^-1^)	8.25 ± 0.71	9.98 ± 2.25	10.28 ± 0.66
TG (mmol L^-1^	3.84 ± 0.56^**b**^	5.56 ± 0.81^**a**^	5.56 ± 0.81^**a**^
AST (U L^-1^)	3.36 ± 1.30	1.53±0.23	1.43 ± 0.34
ALT (U L^-1^)	2.39 ± 0.57	2.27 ± 0.95	1.75 ± 0.29
ALB (g L^-1^)	11.45 ± 0.85	12.90 ± 1.35	13.25 ± 0.98
CAT (U ml^-1^)	2.71 ± 0.00	5.04 ± 1.12	4.17 ± 1.71
T-AOC (U ml^-1^)	0.68 ± 0.11	0.69 ± 0.17	0.86 ± 0.11
MDA (nmol ml^-1^)	27.27 ± 2.95 ^**a**^	20.87 ± 2.16^**b**^	24.29 ± 1.2 ^**ab**^

Note: Values are given as mean ± SD (n = 3) and each group show considerable difference (P < 0.05) within distinct superscripts.

Abbreviations: TP, total protein; T-CHO, total cholesterol; TG, triglycerides; AST, aspartate aminotransferase; ALT, alanine aminotransferase; ALB, albumin; CAT, catalase; T-AOC, total antioxidant capacity; MDA, malondialdehyde.

### Intestinal mucosal morphology

**Fig 1:**
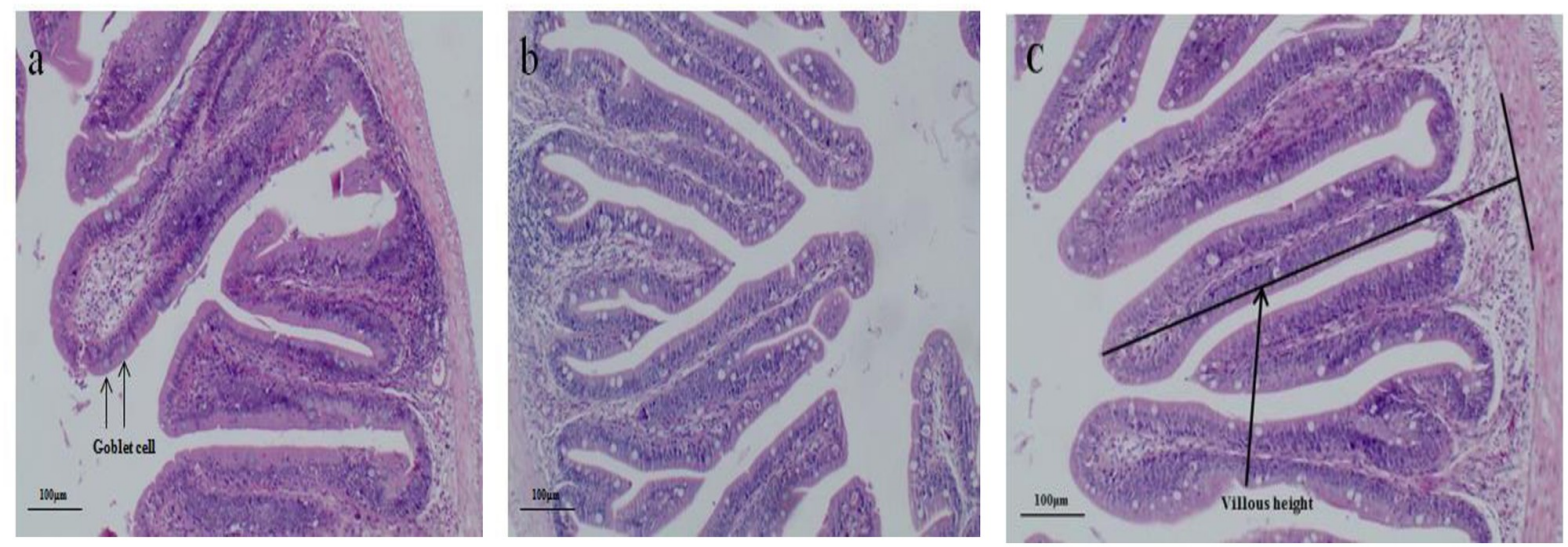
Effects of dietary LA on the structure of fore intestine in juvenile black sea bream (100×) a. Control (0.0) group black anterior intestine villi. b. LA0.1 group (0.1%) black anterior intestine villi: c. LA0.8 group (0.8%) black anterior velvet hair, black arrows marked as villus height and crypt depth.

Table 6 and Fig 1 show the morphology of intestinal mucosa. Villus height and average goblet cell number per villus grew as the percentage of fish fed with LA supplemented diet climbed from 0.0–0.1% and then plateaued.

**Table 6:** The effects of varying levels of lauric acid (LA) in the food on the anatomy of the fore intestinal mucosa in *Acanthopagrus schlegelii*.

Parameters	Diets		
	Control (0.0)	LA1 (0.1)	LA2 (0.8)
Villus height (μm)	384.10 ± 14.208^b^	461.850 ± 15.139^a^	431.213 ± 431.213^a^
Number of goblet cells / villus height	14.090 ± 1.705^b^	27.376 ± 4.041^a^	20.750 ± 2.784^ab^

Note: Values are given as mean ± SD (n = 3) and each group show considerable difference (P < 0.05) within distinct superscripts.

### Microbiota community composition and relative abundance analysis

#### Phylum level

**Fig 2:**
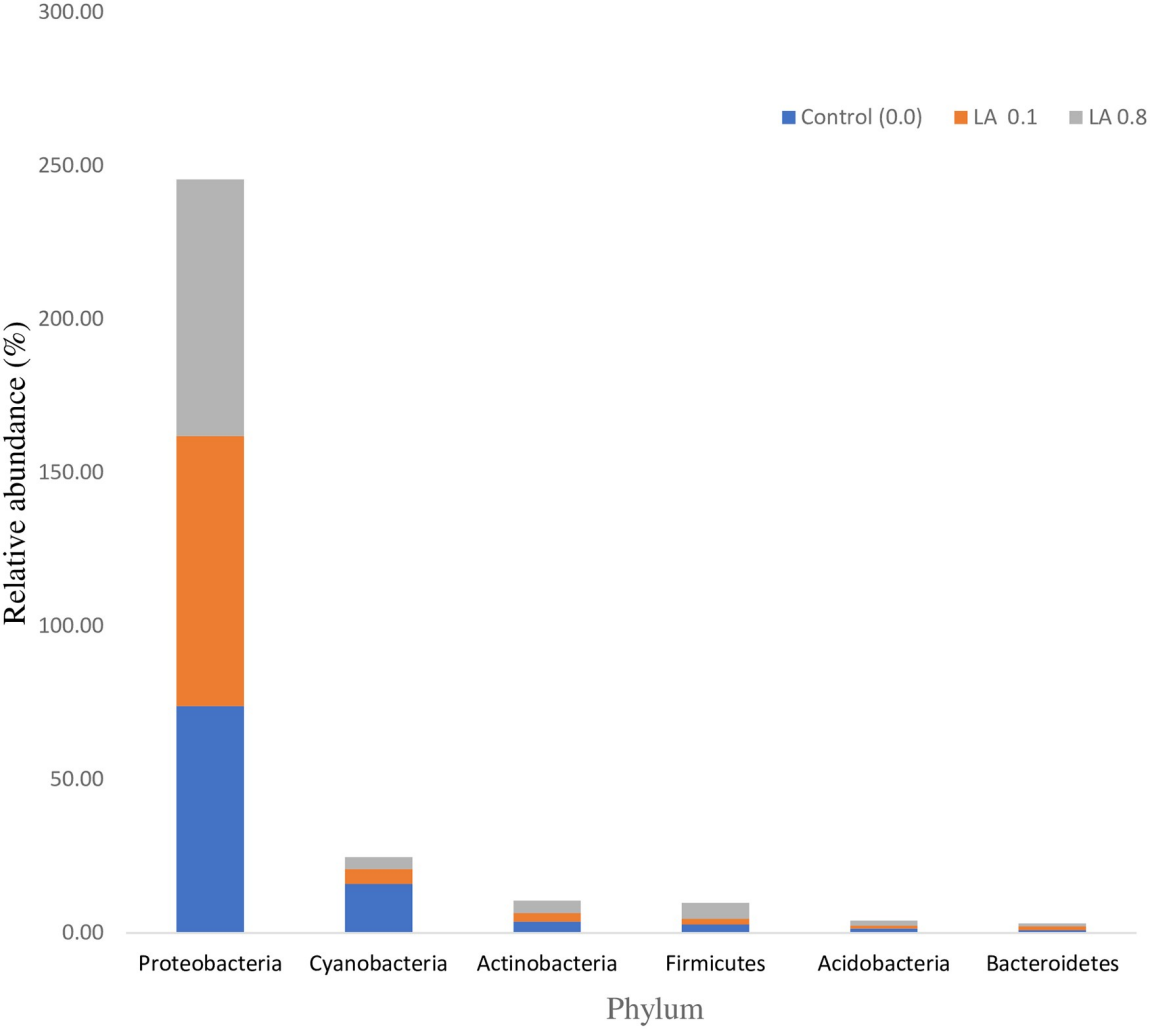
The relative abundance of gut microbiota at the phyla level of black sea bream (n = 4/group).

The microbiota composition at the phylum level in the intestine of black sea bream is represented in Table 7 and Fig 2. In total, 6 phyla were detected in the intestines of the fish fed different dietary treatments. The Proteobacteria, Cyanobacteria, Actinobacteria, Acidobacteria, and Bacteroidetes of phyla were not affected significantly, (P > 0.05) in all dietary groups. Overall, the Firmicutes increased with a higher amount of LA supplementation (P < 0.05).

**Table 7:** The microbiota composition at the phylum level in the intestine of juvenile black sea bream *Acanthopagrus schlegelii* (%).

Phylum	Diets		
	Control (0.0)	LA 0.1	LA 0.8
Proteobacteria	74.01 ± 11.71	87.91 ± 10.43	83.62 ± 2.79
Cyanobacteria	16.01 ± 10.24^a^	4.82 ± 5.09^b^	3.83 ± 0.84^b^
Actinobacteria	3.77 ± 0.35	2.76 ± 2.29	4.00 ± 2.00
Firmicutes	2.85 ± 0.85^b^	1.81 ± 1.60^b^	5.20 ± 0.96^a^
Acidobacteria	1.43 ± 0.92	1.00 ± 1.02	1.61 ± 0.52
Bacteroidetes	0.91 ± 0.50	1.23 ± 0.94	0.98 ± 1.08

Note: Values are given as mean ± SD (n = 3) and each group show considerable difference (P < 0.05) within distinct superscripts.

#### Class level

**Fig 3:**
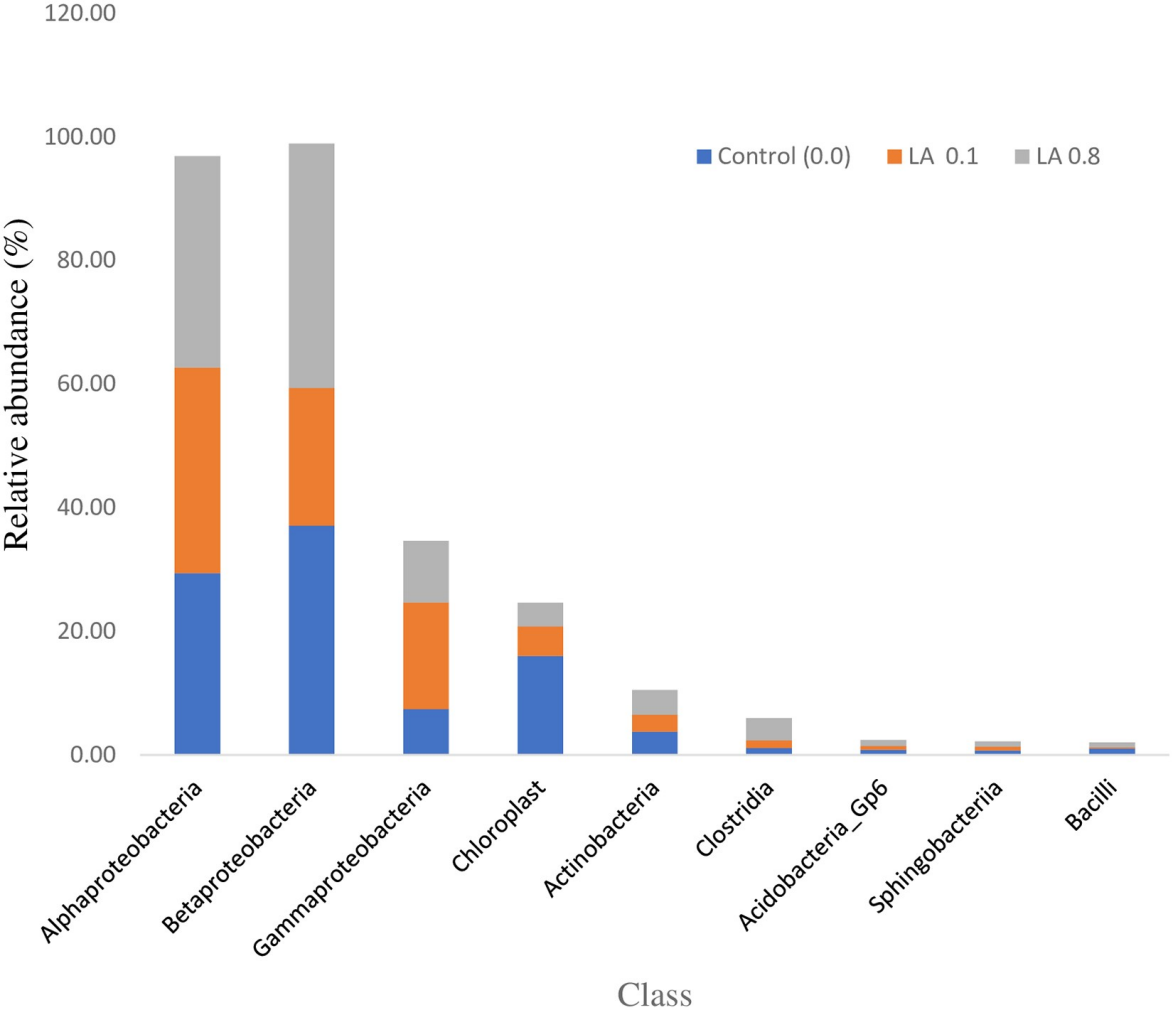
The relative abundance of gut microbiota at the class level of black sea bream (n = 4/group).

The microbiota composition at the class level is represented in Table 8 and Fig 3. In total, 9 classes were detected in the intestines of the fish fed dietary treatments. The Alphaproteobacteria, Chloroplast, Actinobacteria, Acidobacteria_Gp6, Sphingobacteriia, of classes were not affected significantly, (P >0.05) in all dietary groups. Overall, the Betaproteobacteri, Gammaproteobacteria and Clostridia, increased with a higher amount of LA supplementation (P < 0.05). While the Bacilli were reduced with a higher amount of LA supplementation (P < 0.05).

**Table 8:** The microbiota composition at the class level in the intestine of *Acanthopagrus schlegelii* (%).

Class	Diets		
	Control (0.0)	LA 0.1	LA 0.8
Alphaproteobacteria	29.43±7.47	33.29±23.49	34.17±3.17
Betaproteobacteri	37.13 ± 8.03^a^	22.28 ± 9.20^b^	39.49 ± 1.75^a^
Gammaproteobacteria	7.42 ± 4.41^b^	14.33 ± 2.19^a^	9.96 ± 1.25^ab^
Chloroplast	16.01 ± 10.24	4.81 ± 5.01	3.83 ± 0.84
Actinobacteria	3.77 ± 0.35	2.75 ± 2.29	4.00±2.00
Clostridia	1.15±0.41^b^	1.22±1.02^b^	3.59±1.06^a^
Acidobacteria_Gp6	0.87 ± 0.54	0.59 ± 0.54	0.99 ± 0.47
Sphingobacteriia	0.76 ± 0.299	0.62 ± 0.50	0.84 ± 1.14
Bacilli	1.10 ± 0.42^a^	0.15 ± 0.15^b^	0.80 ± 0.54^ab^

Note: Values are given as mean ± SD (n = 3) and each group show considerable difference (P < 0.05) within distinct superscripts.

#### Family level

**Fig 4:**
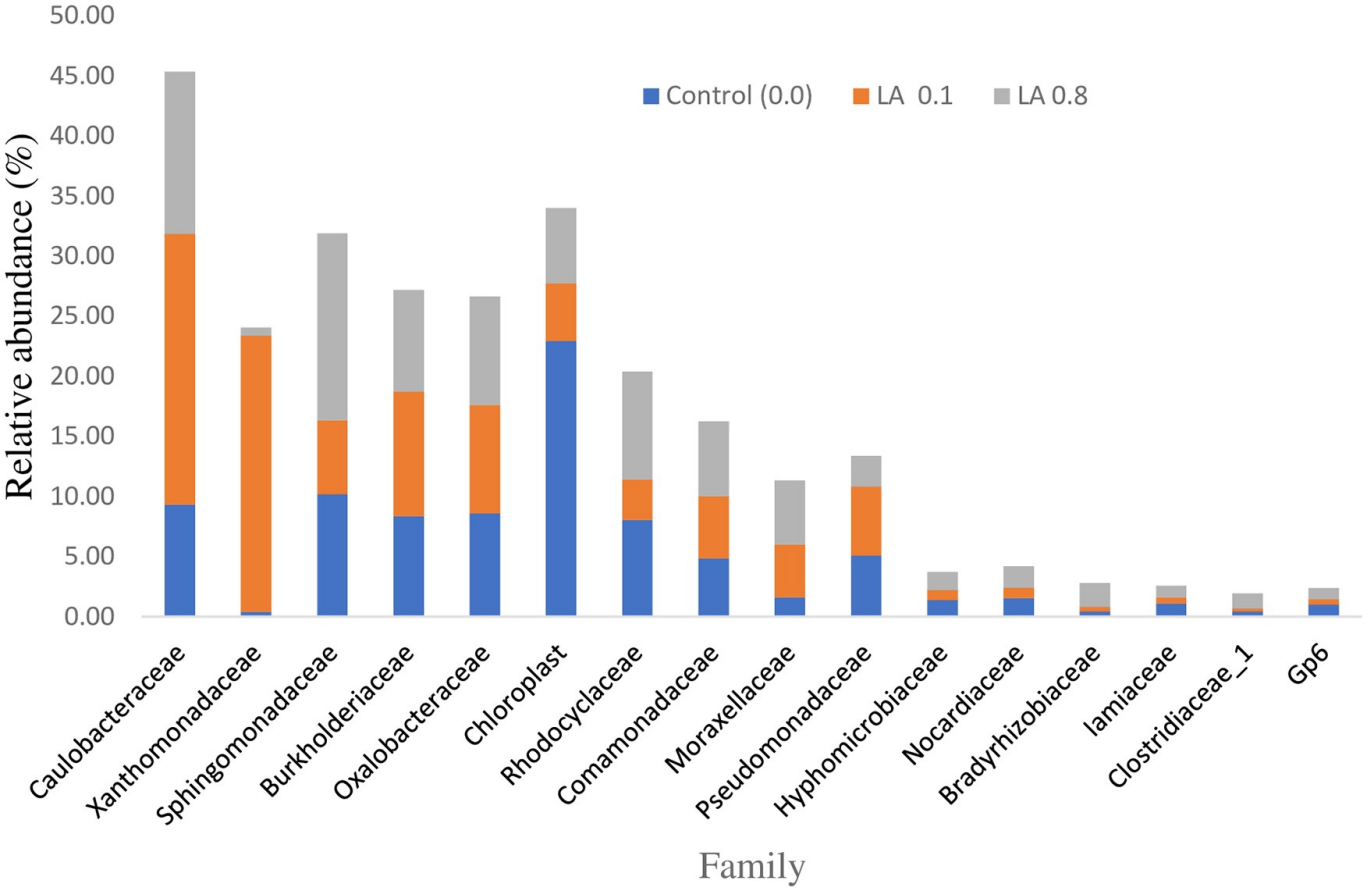
The relative abundance of gut microbiota at the family level of black sea bream (n = 4/group).

The microbiota composition at the family level is represented in Table 9 and Fig 4. In total, 16 families were detected in the intestines of the fish fed dietary treatments. The Caulobacteraceae, Xanthomonadaceae, Sphingomonadaceae, Burkholderiaceae, Oxalobacteraceae, Chloroplast, Rhodocyclaceae, Comamonadaceae, Moraxellaceae, Pseudomonadaceae, Hyphomicrobiaceae, Nocardiaceae, Bradyrhizobiaceae, Iamiaceae and Gp6 of families were not affected significantly, (P > 0.05) in all dietary groups. Overall, the Clostridiaceae increased with a higher amount of LA supplementation (P < 0.05).

**Table 9:** The microbiota composition at the family level in the intestine of *Acanthopagrus schlegelii* (%).

Family	Diets		
	Control (0.0)	LA 0.1	LA 0.8
Caulobacteraceae	14.40 ± 10.22	19.46 ± 21.46	12.46 ± 3.30
Xanthomonadaceae	0.28 ± 0.22	0.11 ±0.09	1.00 ± 1.20
Sphingomonadaceae	9.81 ± 1.98	10.27 ± 8.91	14.04 ± 2.15
Burkholderiaceae	10.25 ± 2.68	7.58 ± 3.31	9.94 ± 2.77
Oxalobacteraceae	12.85 ± 7.43	4.72 ± 4.43	9.11 ± 2.65
Chloroplast	16.01 ± 10.24	4.81 ± 5.01	3.83 ± 0.84
Rhodocyclaceae	7.03 ± 3.47	5.11 ± 5.10	10.56 ± 2.80
Comamonadaceae	5.54 ± 0.95	4.54 ± 1.65	6.88 ± 1.39
Moraxellaceae	1.04 ± 0.72	7.37 ± 7.50	3.33 ± 2.37
Pseudomonadaceae	4.38 ± 3.03	5.37 ± 6.70	2.29 ± 1.28
Hyphomicrobiaceae	1.31 ± 0.17	0.95 ± 0.69	1.95 ± 0.64
Nocardiaceae	1.63 ± 0.23	0.99 ± 1.36	1.62 ± 1.08
Bradyrhizobiaceae	0.45 ± 0.08	0.63 ± 0.39	1.94 ± 1.79
Iamiaceae	0.96 ± 0.49	0.73 ± 0.64	1.12 ± 0.87
Clostridiaceae	0.31 ± 0.21^b^	0.79 ± 0.64^ab^	1.52 ± 0.38^a^
Gp6	0.87 ± 0.54	0.59 ± 0.53	0.99 ± 0.47

Note: Values are given as mean ± SD (n = 3) and each group show considerable difference (P < 0.05) within distinct superscripts.

## Discussion

Diets containing medium-chain fatty acids (MCFAs) have advantages over other feed because it is rapidly digested, can be passively absorbed by the animals [8]. In the current study, the weight gain rate, hepatosomatic index (HSI), growth performance and specific growth rate of juvenile black sea bream were increased due to feeding upon LA supplemented diets. Similar results were obtained by using caprylic and capric acids on piglets comparing the influence of dietary supplements of 0.2% [30]. Hong, Hwang [31] reported that caproic and caprylic MCT (1:1 ratio), increased body weight in piglets. The possible mechanism of the reduced intake of food is the reduction of ghrelin by MCT, which is released from gastric mucosa and has a role in increased food intake [32]. This effect might be induced by the acetylation of Ghrelin by MCFA and MCT that alters its activity [33]. Similar results were found for coconut oil which can induce such effects by antimicrobial activity in the alimentary canal that induced digestive functions [34]. Additionally, MCFA is recognized to raise metabolism [35]. Further, the controlled release of MCFA from CO suppresses the microorganisms in the intestinal region (total anaerobic count, lactobacilli, *E*. *coli*) and improves the mucosal health in piglets [36]. Previously, it was reported that distilled medium-chain fatty acids of coconut oil improved weight gain by increasing food intake in fish [21]. Similarly, the addition of organic acid in the diet stimulated fish growth which is directly dependent on the type of organic acid, diet composition, fish species as well as age, and condition of farming [37]. Dierick, Decuypere [36] report that both free MCFA and bound MCFA with triglyceride (2.5%) in a piglet diet-induced gain in body weight and FE than control animals which were fed with soybean oil. A supplement of LA was non significantly improving the proximate composition of the whole body and dorsal muscle. This diet can induce such an effect without secretion of cholecystokinin by MCFA in the region of piglets small intestine [38].

To digest food materials in the digestive tract, a variety of enzymes play a crucial role in the digestion of food materials that can in turn cause an increase in weight gain and better health in fish. Digestive enzymes activities estimation is useful to determine the nutrient assimilation ability of fish in a given diet [39]. The dietary treatments in digestive enzymes find (lipase, amylase and trypsin) no significant change in the stomach, foregut, midgut and hindgut but significant increase lipase activity in the foregut region. Similar results were reported previously, that *Cuphea* seeds supplementation can induce lipase activity and further increase the height of villus in piglets when they were supplied seeds of *Cuphea* and with supplementation of MCFA as a source of lipase (9).

To determine the better health and nutritional uptake in black sea bream fingerlings supplied with the LA, several immunes, biochemical and antioxidant parameters were determined in this study. These parameters included TP, T-CHO, TG, AST, ALT, ALB, CAT, T-AOC and MDA. These parameters indicated a positive effect of LA 0.1% supplementation on the physiology of the fish as well as on the immunity in the treated groups. In previous studies, similar results were observed in poultry farming using different breeds [40] and a more robust immune system [41]. The higher TP in the present study indicates that protein metabolism is better [42] while higher TG in the control group shows that the lipid has been excessively formed in the body and is deposited in the liver which can adversely affect the health of the organism [43, 44].

Related to serum biochemical parameters, AST, ALT and ALB were not significantly changed, showing that that LA exposure did not cause a substantial impact on the kidney and liver. Alteration in the levels of AST and ALT in the serum indicates hepatocellular damage [45] which were not shown in the present study. Similar results were reported previously in rats treated with virgin coconut oil [46]. Antioxidant enzymes were determined in the present study to determine oxidative stress within the tissues of the fish. CAT studied various mechanisms to decrease H_2_O_2_ to H_2_O and O_2_. In this mechanism, SOD generates less amount reactive ROS during the process of O2 dismutating [47]. T-AOC includes antioxidants (enzymatic and non-enzymatic) and is used to determine the protection against oxidative stress in the animal’s body [48]. According to the results, the LA affected liver CAT activities and T-AOC content suggesting that LA can induce antioxidants enzymes content in the body that can fight against the ROS in return. Our results are in agreement with Seneviratne and Sudarshana Dissanayake [49, 50] suggesting that some compounds in coconut oil (LA and MCFA), when used as food supplements can improve antioxidant enzymes that can fight diseases caused by oxidative stress [51]. MDA is a lipid peroxidation product and is frequently measured as an oxidative stress marker [52]. In the current study, the MDA amount in the serum was significantly decreased. Consistent with our results, [53] reported that malic and citric acids, have antioxidant activities. Although very limited data are available on aquatic animals, however, the mentioned literature support the current findings: LA supplementation improved enhance immunity, serum biochemical and antioxidant indicators in black sea bream (*A*. *schlegelii*).

In the current investigation intestinal mucosal morphology height of villus and goblet cells, number/villus height increased in LA supplemented diet from 0.0% to 0.1% suggesting that dietary supplements have played their role in increasing the height of villus in the intestine. In previous studies it was reported that organic acids are used as a food supplement, it can cause a significant increase in villus height [54, 55]. In animals, the intestinal villus plays a vital role in food materials absorption and digestion as well as helps in boosting the immunity of an organism by providing a home to microbiota [56]. It contains the epithelial cells that play a role in digestion and absorption while the goblet cells produce mucous that protect the underlying layers as well as lubricate the food materials and are also serving as a transport medium for various materials across the layers [57, 58]. These reports show a positive correlation between weight gain and villus health in piglets [59]. Greater is the villus height. It will be the protection provided to the epithelial cells; it will also be the transport of materials across the layer. This integrity can also be improved by food supplements like soybean meal in the diet [60].

In this study intestinal mucosal morphology height of villus and goblet cells number/villus height increased in LA supplemented diet from 0.0% to 0.1%. A similar result has been reported that organic acids in all diets may increase villus height [54, 55].

In the present study, improvement in villus height was observed in fish provided with LA supplements in the diet. In previous studies, MCT supplements piglets induced villus length crypt ratio as well as activity [7]. In another study, it was reported that MFCA as a food supplement can have similar effects in the intestine of broilers suggesting that MFCA can a direct source of energy for the enterocytes [61]. Similar results were reported by Takase and Goda [62] suggesting that food supplement MCTs can improve the morphology of the intestine by increasing phospholipid/ protein ratio, enlarging intestinal villi, reducing crypts, and enhancing membrane-bound enzymes activity. Alike results were attained in the current study that LA exerts a positive influence on the structure of intestinal mucosa in juvenile black sea bream.

Similar results of Dierick, Decuypere [9] were also reported in swine using MCFAs; a significant enhancement in the length of villi of small intestine collectively with a lower side of crypt depth as well as intraepithelial lymphocytes with a lower number was observed. Hanczakowska, Szewczyk [30], previously reported that caprylic as well as capric acids nourished together or separately significantly enhanced piglet body weight and improved villus height.

In an animal, microbiota in the gut plays a vital part in animal health. If the microbiota in the gut is harmful, it can result in a variety of complications like obesity, a metabolic syndrome that is possibly due to toxic substances produced by the bacteria which can increase the intestinal permeability for LPS into the blood and can cause an inflammation state in the body [9, 24]. In the current investigation, dietary supplementation of LA effect, the intestinal microbiota composition. The Proteobacteria, Cyanobacteria, Actinobacteria, Firmicutes, Acidobacteria, and Bacteroidetes of phyla were detected in the dietary groups. However, Firmicutes increased with elevated concentrations of LA supplementation. Our findings are following previous findings in fish species, it was reported that fish gut contained Firmicutes and Proteobacteria as the most dominant microbes. The population of these phyla were independent of the diet [63]. In another study, it was found that the Proteobacteria population in the gut was dominant [64]. The reason behind this is that generally, the dominant phylum about 70% Firmicutes are a transient microbial community in the distal part of the intestine [63]. These bacteria are considered beneficial and usually used as probiotics for vertebrates especially fishes. If the number of these microbes is high then it is considered desirable as some of the individuals of these phyla are involved in the conversion of indigestible carbohydrates into digestible ones as well as fibers utilization in energy production is desirable [65, 66]. However, in the current work, the Betaproteobacteri, Gammaproteobacteria and Clostridia are significantly higher than the control group. In the current investigation, 0.1% of LA is enough to increase the extent of gut microbiota in the intestinal region of black sea bream which further supports the propagation of Firmicutes. Generally, plant diets are directly related to the high amount of gut microbiota [11, 63]. A higher number of Gammaproteobacteria indicates the use of plant diets and helping in degrading cellulose. On the other hand, an increase in the Proteobacteria shows a modification in the balance of gut microbiota. Intestinal microbiota imbalanced could negatively affect the intestine immune system that improves diseases development in fish [66]. Our data revealed that dietary LA supplemented in the diet was related with a large amount of gut microbiota in comparison with control diet.

A similar trend was previously reported in fish following dietary administration of SILOhealth 108Z [67]. Remarkably, Kollanoor, Vasudevan [68] Caprylic acid as well as its monoglyceride constituent which are present both in short and medium-chain 1-monoglycerides of SILO health 108Z mix have been shown to have in vitro antibacterial action against Edwardsiella species belonging to the Gammaproteobacteria class. Our findings are consistent with GML treatment and the gut microbiota, both of which have an influence on host health and physiology, notably metabolism and immunological development [9, 24].

## Conclusion

In summary, the proper amount of LA dietary supplementation in the fish feed can significantly enhance the growth performance, digestive enzymes, feed efficiency, serum immune index and antioxidant capacity of juvenile black sea bream. The current findings suggested thatLA is very useful and 0.1% level is the best dose in our study. Further, the addition of LA to the diet can significantly increase villus height and number of goblet cells per villus height of the anterior intestine and as well as gut microbiota in juvenile black sea bream. Molecular based studies of the intrinsic links between the regulatory pathways and the physiological functions are suggested for further clarification of the actual mechanisms of LA.
